# Diagnostic Value of Bacteria

**Published:** 1899-04

**Authors:** Wm. L. Morgan

**Affiliations:** Baltimore, Md.


### DIAGNOSTIC VALUE OF BACTERIA.

By Wm. L. Morgan, M. D., Baltimore, Md.

April 8th, 1898, Miss E. M., age 20, four years ago had a long sick spell, called typhoid fever, and for several weeks took drugs to the satisfaction of allopathic science, especially Arsenic, Quinine, Iron, and Strychnine. When I received a description of her case and the treatment, which was a complete case of ex-ophthalmic goitre, I at once sent Calc-carb., CM. and S. L., which cured her in a few weeks.

Has all her life been subject to attacks of sore throat, otherwise good health. Has been a student at the State Normal School of the State of West Virginia, to the above date, when she started with 150 others of the school on an excursion to Washington, D. C. Going eastward through the Allegheny Mountains from current of air in cars took cold. On arriving in Washington had some fever, headache, and a sore throat, common with her. A doctor was called, and applied the usual amount of swabbing and drugging. I saw her on the 10th, late in the afternoon. I found her in a hotel, in a small room, with one window and two beds. Four girls had been rooming with her for two nights, and scores of the party were visiting her. The doctor had taken a culture and sent it to the city laboratory, where it was pronounced a true case of diphtheria, with true germs. They had ordered quarantine to be established.

There was a pitted or eating ulcer on left tonsil, very sore, worse after sleeping. I gave her Lach., CM. The next day she was much better, but she must go to the hospital by decree of the health officers. The ambulance was a tight, suffocating conveyance. I left her in the hospital feeling worse from the debilitating ride.

On the 13th I saw her again. The sore had spread to uvula and nasal cavity, and looked yellow. Gave Kali-bichro., CM., and engaged Dr. J. B. G. Custis to manage the case, as

I could not go as often as necessary. He gave Apis, which did well for a few days, and afterward, as improvement seemed slow, a dose of Sulphur finished the cure. On the 24th Dr. Custis wrote me she was well, but could not go out, as there were still germs in the culture.

I at once wrote to her father, directing him to make diligent inquiry if any of the excursionists had taken the disease and to answer by telegraph. The second day a dispatch came: "Investigated carefully; not one sick in any way since."

May 24th.—Went to see patient. Her throat was entirely well and had been for twelve days. No trouble but the bad effects of close confinement.

I was informed by the matron that she would have to stay four weeks after the throat was well, or until the germs were gone, which meant two weeks more. I answered that my contract had now been out two weeks, but that I would pay up to the present, and demand her release. After some hints at my lack of honor in not allowing this to go on, the money was taken, and I went home. I then placed the case in the care of a lawyer, with instructions to proceed. He at once opened correspondence with her father, 300 miles away, in West Virginia.

On the morning of May 9th Miss M. was informed that germs were still present, and there was no hope of her getting out. At 3 P. M. a telegram came from the West Virginia lawyer through the police department, asking why she was detained so long after she was well, and giving a hint that legal proceedings were commenced for her release. At 5 P. M. she was told to get ready to go on the next train. At 7.30 she was dressed and went out without any disinfection of clothes that had been in the room with her for weeks. The culture I took on the 6th and the one she sent me on the 26th still had microbes, but nobody had ever taken the disease from her, though she had been in school and among people all the time until June 13th.

Some of the evidence the health officers and physicians made capital out of was, that certain of the students remembered that several weeks before a boy in another department of the school had a sore throat. Dr. W. took it for granted that my patient had attended chapel service at the same time, but nobody knew whether the boy had left the school or not, so it amounts to no evidence at all as to contagion.

I do not give this history to exploit homœopathic treatment, but to show what abuses can be made of the germ theory by an arbitrary health officer. In twenty-five years' constant practice and careful investigation of all cases of diphtheria within my reach, I have entirely failed to find a single case where there was any evidence of contagion. I have not had one case that could be proved to have the disease by infection from another, nor has any case of mine ever conveyed the disease to another; but I have met with clear evidence of its coming from bad sanitary surroundings, and from sewer and cesspool gases.

### Discussion.

Dr. Patch—I would like to say a few words about the matter of contagion. Some years ago I had a case of diphtheria that was brought from Nantucket to our town. During the two weeks following the father of the boy acted as nurse, and came down with the disease. He had a long, tedious sickness, resulting in recovery. After a short time a nurse from out of town, who had been called to attend him, also sickened and had to go back to the hospital in Boston. In the cases mentioned after the reading of Dr. Kimball's paper, about ten days after the first case the father was taken sick in the same surroundings, but the youngest child was kept away from the house for some four weeks. After the death and recovery of the first cases everything was taken out of the house and thoroughly disinfected, carpets, curtains, furniture, etc. Then the youngest child was brought back. A few days after she was taken sick with diphtheria, and in about a week died. This seems very much like contagion.

Dr. Morgan—Did you investigate the sanitation?

Dr. Patch—The child had not been in those surroundings, and the nurse had also come from out of town to nurse the boy. The house was isolated and small, but it was new. It had no plumbing. If there was anything bad in the vicinity it came from a marsh back of the house.

Dr. Kennedy—Dr. Morgan does not think that his cases resulted from contagion, but I think there are well authenticated cases that have resulted from contagion. I recall a case where an Alumnus of Boston University had a difficult case of diphtheria under treatment. In his anxiety and efforts to save the child (it being a case where tracheotomy was performed), he sucked the membrane from the throat to save the little one from strangling, and was taken sick immediately and died. It is very singular how this disease works. We find cases where it is high and dry, and where it is low and damp, in the country, by the sea-shore, and in the mountains, so that it is difficult to account for the disease, but I believe that it is often due to unsanitary conditions.

Dr. Pease—I believe one important element to be thought of in considering the problem of contagion is susceptibility. It is demonstrated where one or more of a family have the disease, and other members pass through the entire siege without any symptoms of the disease appearing. I would like to ask Dr. Patch if any persons from without who came in contact with his cases contracted the disease?

Dr. Patch—In the first case no outsiders were admitted, but in the second case I think there were some who came in contact with it and did not have the disease. We had two nurses who went through it without any trouble.

Dr. Pease—There is no doubt that there is a contagious element as well as in other diseases, but there are people who are not susceptible.

